# Imaging diagnosis for chronic lateral ankle ligament injury: a systemic review with meta-analysis

**DOI:** 10.1186/s13018-018-0811-4

**Published:** 2018-05-22

**Authors:** Shengxuan Cao, Chen Wang, Xin Ma, Xu Wang, Jiazhang Huang, Chao Zhang

**Affiliations:** Department of Orthopedics, Huashan Hospital, Fudan University, No.12, Middle Wulumuqi Road, Jingan District, Shanghai, China

**Keywords:** Ankle ligaments, Meta-analysis, Imaging, Diagnostic accuracy

## Abstract

**Background:**

Various imaging techniques have been utilized for the diagnosis of chronic lateral ankle ligament injury. This systemic review will explore the effectiveness of different imaging techniques in diagnosing chronic lateral ankle ligament injury.

**Methods:**

Relative studies were retrieved after searching 3 databases (MEDLINE, EMBASE, and Cochrane Central Register of Controlled Trails). Eligible studies were summarized. Data were extracted to calculate pooled sensitivity and specificity of magnetic resonance imaging (MRI), ultrasonography (US), stress radiography, and arthrography.

**Results:**

Fifteen studies met our inclusion and exclusion criteria. A total of 695 participants were included. The pooled sensitivities in diagnosing chronic ATFL injury were 0.83 [0.78, 0.87] for MRI, 0.99 [0.96, 1.00] for US, and 0.81 [0.68, 0.90] for stress radiography. The pooled specificities in diagnosing chronic ATFL injury were 0.79 [0.69, 0.87] for MRI, 0.91 [0.82, 0.97] for US, and 0.92 [0.79, 0.98] for stress radiography. The pooled sensitivities in diagnosing chronic CFL injury were 0.56 [0.46, 0.66] for MRI, 0.94 [0.85, 0.98] for US, and 0.90 [0.73, 0.98] for arthrography. The pooled specificities in diagnosing chronic CFL injury were 0.88 [0.82, 0.93] for MRI, 0.91 [0.80, 0.97] for US, and 0.90 [0.77, 0.97] for arthrography.

**Conclusion:**

This systematic review with meta-analysis investigated the accuracy of imaging for the diagnosis of chronic lateral ankle ligament injury. Ultrasound manifested high diagnostic accuracy in diagnosing chronic lateral ankle ligament injury. Clinicians should be aware of the limitations of MRI in detecting chronic CFL injuries.

## Background

Ankle sprain is one of the most common sports injuries [1–3]. The injury mechanism, a combination of inversion and adduction in foot plantar flexion, can cause damage to the lateral ankle ligaments [4]. Lateral ankle ligaments comprise anterior talofibular ligament (ATFL), calcaneofibular ligament (CFL), and posterior talofibular ligament (PTFL) [5]. Chronic lateral ankle ligament injury is a long-term outcome in patients suffering acute ankle sprain. Some long-term cohort studies showed that 10 to 20% patients eventually developed persistent symptoms, including pain, swelling, perception of instability, and recurrent ankle sprain [6–9]. The precise cause of these symptoms is still in debate and probably multifactorial [5, 10–12]. But identifying chronic lateral ankle ligament injury is critical for locating primal symptoms, and thus, the decision making of surgical intervention [13].

Compared to acute ligament injury, the proper diagnosis of chronic ligament injury is difficult because of the uncertain association between initial inversion trauma history and ligament injury and diversified manifestation of chronic injuries on images [14, 15]. The patients’ history and clinical tests are important in diagnosis. A meta-analysis discussed the accuracy of clinical tests in diagnosing ankle ligament injury and claimed that clinicians cannot rule out ligamentous injury when physical tests are negative, and additional imaging is warranted [16]. Imaging is a helpful diagnostic method according to consensus of experts [17, 18]. However, different from acute injury, chronic ligament injury could show as either stretching, rupture, scarring, or thickening of the ligament on images, which confuses the clinical practitioners [19]. Magnetic resonance imaging (MRI), ultrasonography (US), stress radiography, and arthrography are often utilized for diagnosing chronic lateral ankle ligament injury. Separate studies on various imaging diagnostic techniques have been reported; however, the diagnostic effectiveness of different imaging techniques was still controversial, partly due to different existing reference standards [20, 21].

A previous systematic review assessed US for diagnosis of chronic ankle instability, but utilized variable gold standards, and did not provide pooled data [20]. Studies of MRI, US, stress radiography, and arthrography for diagnosis of chronic lateral ankle ligament injury were reported, but no previous systemic review provided synthesized data. This systemic review with meta-analysis is trying to analyze studies on diagnostic accuracy of different imaging techniques on chronic lateral ligament injury. Arthroscopic or surgical findings are set as the gold standards of ligament injuries [12, 22].

## Methods

### Inclusion and exclusion criteria

The studies that met the following criteria were included: (1) cohort-type or cross-sectional studies; (2) evaluated MRI and/or US and/or stress radiography and/or arthrography for the diagnosis of chronic ATFL and/or CFL injury (regarding the classification of acute and chronic ligament injuries, we followed the decisions adopted by the authors of the studies included); (3) comparing imaging results with arthroscopic or surgical findings as reference standards, and (4) reported data that enabled the calculation of the number of true positive (TP), true negative (TN), false positive (FP), and false negative (FN).

The following criteria were used to exclude underqualified studies: (1) acute injury patients; (2) patients with confounding factors like ankle fracture, history of previous foot, and ankle surgeries; (3) without clearly described arthroscopic or surgical findings as their reference standards; (4) cadaveric studies or studies utilizing animal models; and (5) non-English articles.

### Search strategy

A systematical literature search was conducted to include the following three databases: MEDLINE, EMBASE, and Cochrane Central Register of Controlled Trails (CENTRAL). The detailed search strategies (Table 1) were first developed in MEDLINE and were then adjusted and applied in the other two databases.

**Table 1:** Detailed search strategies

Step	MEDLINE	EMBASE	CENTRAL
1	accuracy[Title/Abstract] OR accurate rate[Title/Abstract] OR diagnostic value[Title/Abstract]	exp diagnostic accuracy/ or exp accuracy/ or exp measurement accuracy/ or exp. diagnostic test accuracy study/	MeSH descriptor: [Sensitivity and Specificity] explode all trees
2	sensitivity and specificity[MeSH Terms]	sensitivity.mp.[mp=title, original title, abstract, name of substance word, subject heading word]	accuracy: ti, ab, kw or accurate rate: ti, ab, kw or diagnostic value: ti, ab, kw(Word variations have been searched)
3	chronic ankle instability OR functional ankle instability OR lateral ankle instability	specificity.mp.[mp=title, original title, abstract, name of substance word, subject heading word]	MeSH descriptor: [Lateral ligament, Ankle] explode all trees
4	PTFL OR CFL OR ATFL	exp ankle ligament/	PTFL OR CFL OR ATFL(Word variations have been searched)
5	talofibular[Text Word] OR calcaneofibular[Text Word] OR posterior talofibular[Text Word] OR anterior talofibular[Text Word]	ATFL.mp.[mp=title, original title, abstract, name of substance word, subject heading word]	chronic ankle instability OR functional ankle instability OR lateral ankle instability(Word variations have been searched)
6	lateral ligament, ankle[MeSH Terms]	CFL.mp.[mp=title, original title, abstract, name of substance word, subject heading word]	talofibular OR calcaneofibular OR posterior talofibular OR anterior talofibular(Word variations have been searched)
7	(1 OR 2) AND (3 OR 4 OR 5 OR 6)	PTFL.mp.[mp=title, original title, abstract, name of substance word, subject heading word]	(1 OR 2) AND (3 OR 4 OR 5 OR 6)
8		exp ankle instability/	
9		(1 OR 2 OR 3) AND (4 OR 5 OR 6 OR 7 OR 8)	

*Abbreviations*: *exp* explode, *ti* title, *ab* abstract, *kw* key words

Retrieved articles from each database were at first screened for duplication. Then, after titles and abstracts screening, relevant studies for this systemic review underwent full-text screening. Eligible studies were included according to the aforementioned inclusion and exclusion criteria.

### Data extraction and quality assessment

The extracted data include authors, publication years, demographic features of participants, study design, index tests, gold standards, and the numbers of true positive, false negative, false positive, and true negative subjects. The pathologic features of chronic injury lead to various manifestations on images [14, 19, 23]. We eliminated this diversity by dichotomized imaging results to “injured” and “intact” in this study for better comparability among different included studies. Different kind of injuries such as “stretching,” “rupture,” “scarring,” or “thickening” are all categorized as “injured.”

Two authors independently extracted these data and filled previously drafted forms for this review. Results of the two authors were cross-validated, and discrepancies were mediated by the third author. The quality of the included articles was assessed through revised Quality Assessment of Diagnostic Accuracy Studies (QUADAS-2) tool, a widely recommended scale for diagnostic test evaluation [24]. According to QUADAS-2 tool, risk of bias was assessed in terms of patient selection, index test, and reference standard.

### Statistical analysis

Sensitivity and specificity of each index test in individual study were calculated in Meta-DiSc, version 1.4.0, using the extracted data of TP, FN, FP, and TN. Pooled sensitivity and specificity were calculated using the total number of TP, FN, FP, and TN subjects in all relevant studies. Likelihood ratio (LR) evaluates the discriminatory properties of the test results [25]. Positive and negative likelihood ratio evaluates the positive and negative test results respectively. Pooled positive and negative likelihood ratio was calculated using random effects model. The diagnostic odds ratio (DOR) is defined as (true positives × true negatives) / (false positives × false negatives), which evaluates the overall diagnostic test performance combining sensitivity, specificity, and likelihood ratio [26]. Pooled DORs were calculated using random effects model.

All final outcomes were presented with 95% confidential interval. Pooled sensitivity, specificity, likelihood ratio, and DOR are calculated concerning each subgroup. Heterogeneity testing was assessed using the *I*^2^ test. A value of *I*^2^ > 50% was considered to be significant heterogeneity among the pooled data.

## Results

### Description of included studies

**Fig. 1:**
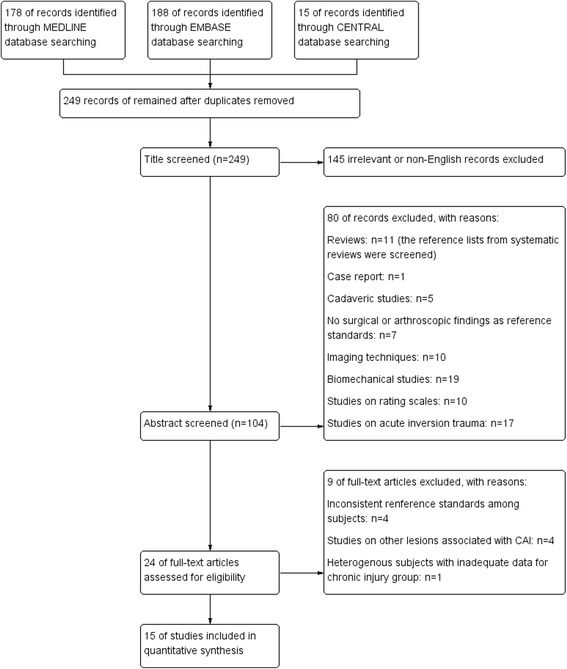
Flow diagram of search strategy

A total of 178 articles were retrieved from MEDLINE. One hundred eighty-eight articles were retrieved from EMBASE. Fifteen articles were retrieved from Cochrane Central Register of Controlled Trails. After deleting duplications, a total of 249 studies were identified in the primary search of three aforementioned databases. Then, these studies were screened for eligible studies as presented in the flow chart (Fig. 1). Twenty-four studies underwent full-text screen, and 9 studies were excluded for the following reasons: inconsistent reference standards among subjects [21, 27–29], studies on other lesions associated with chronic ankle instability [30–33], and heterogeneous population with inadequate data for chronic injury group [34].

Overall, 15 studies [35–49] were eligible for this systemic review and are summarized in Table 2. A total of 695 participants were included. In 3 studies [39, 45, 49], participants were divided into acute injury group and chronic injury one, chronic injury groups of which were included in data synthesis. Two studies [42, 44] reported inter-rater reliability. In one study [48], a number of ankles instead of a number of participants were considered.

**Table 2:** Summary of included studies

Study (author and year)	Subject features	Age	Gender	Time from injury to study commencement/duration when patients presented with symptoms	Study design	Level of evidence	Index tests	Gold standard	Main conclusion
Cha et el. 2012	65 patients diagnosed with chronic lateral ankle instability who received preoperative MRI and arthroscopic examination	Not mentioned	39 men and 26 women	Not mentioned	Case-control study	3b	MRI	Arthroscopic findings	“However, inter-observer reliability and sensitivity of MRI findings were found to be relatively low in this study. Therefore, arthroscopic examination before ligament repair is a useful and recommended method for diagnosis and treatment of intra-articular lesions.”
Chandnani et al. 1994	17 chronic ankle instability patients	24.5 years (ranged from 20 to 48 years)	14 men and 3 women	Presented with symptoms of at least 6-months duration and did not respond to conservative therapy that included immobilization and early aggressive rehabilitation	Cohort study	2b	Stress radiograph, MRI and MR arthrography	Findings from either diagnostic arthroscopy (*n* = 3) or open surgical repair (*n* = 14)	“In conclusion, our results suggest that MR arthrography may be a sensitive technique for detecting and staging tears of the lateral collateral ligaments and evaluating associated injuries in patients with chronic ankle instability. In patients for whom surgery is contemplated, MR arthrography may be a useful adjunct to conventional imaging techniques.”
Cheng et al. 2014	120 patients with a clinical suspicion of chronic ankle ligament injury	32 years (ranged from 15 to 70 years)	85 men and 35 women	Duration of symptoms ranged from 6 weeks to 20 years (mean, 2.2 years)	Cohort study	2b	Ultrasonography	Arthroscopic findings (42 of these patients also underwent open surgery)	“In conclusion, ultrasonography as a convenient technique with low costs and real-time examination showed a satisfactory sensitivity and specificity for detecting lateral ligament injury.”
Cho et al. 2016	28 consecutive lateral ankle instability patients who underwent ankle arthroscopy	32.4 ± 11.9 years (ranged from 15 to 55 years)	19 men and 9 women	Conservative treatment had failed to substantially alleviate the symptoms for at least 3 months	Case-control study	3b	MRI, manual anterior drawer test, stress ultrasonography, and stress X-ray	Arthroscopic findings	“Manual stress US is as precise as MRI in the detection of ligament injury and provides information on chronic ankle instability.”
Chou et al. 2006	50 patients suffering from ankle instability (17 of them who would receive surgical intervention were included in this review.)	Ranged from 14 to 77 years, *n* = 50	29 men and 21 women, *n* = 50	Not mentioned	Cohort study	2b	MRI and MR arthrography	Surgical findings	“For evaluating ankle disability, using plain MRI alone is not adequate for correctly detecting lateral collateral ligamentous injury of the ankle joint. MR arthrography improves the sensitivity and the accuracy for ATaF and CF ligament injuries. It also helps in assessing coexisting pathologic lesions of ankle joints.”
Hua et al. 2012	83 consecutive patients underwent ankle arthroscopy for different diagnosis	32.2 years (ranged from 17 to 57 years)	51 men and 32 women	Not mentioned	Case-control study	3b	Ultrasonography	Arthroscopic findings	“US examination is a reliable and accurate method to evaluate chronic ATFL injury.”
Joshy et al. 2010	24 patients underwent arthroscopy as well as MRI for chronic ankle pain and/or instability	39 years (range from 11 to 65 years)	12 men and 12 women	Not mentioned	Case-control study	3b	MRI	Arthroscopic findings	“MRI scan has very high specificity and positive predictive value in diagnosing tears of ATFT, CFL and osteochondral lesions. However sensitivity and negative predictive value is low with MRI.”
Kim et al. 2015	79 patients who would undergo ankle arthroscopy for different diagnosis.	34.6 years (ranged from 21 to 67 years)	44 men and 35 women	Mean duration of symptoms was 25.9 weeks (range, 11 to 52 weeks)	Cohort study	2b	MRI	Arthroscopic findings	“The specificity and positive predictive value of MRI in the diagnosis of ATFL injuries were very high, whereas the sensitivity and negative predictive value of MRI were relatively low.”
Kumar et al. 2007	58 patients with symptomatic instability of their ankle	28 years (ranged from 18 to 50 years)	47 men and 11 women	Unresponsive to physiotherapy and non-operative management for at least 6 months	Cohort study	2b	MRI and stress radiography	Arthroscopic findings	“MRI did not demonstrate any distinct advantage over the examination under anesthesia and stress radiography in the diagnosis of grade III lateral ankle ligament injuries.”
Lee et al. 2012	34 patients who underwent ankle MRI because of ankle sprains or ankle instability	29 years (ranged from 13 to 53 years)	22 men and 12 women	Not mentioned	Cohort study	2b	MRI	Arthroscopic findings	“|A cortical defect with a bright dot-like or curvilinear high-signal-intensity lesion on T2-weighted MRI may be regarded as an additional finding to help increase the diagnostic performance for detecting anterior talofibular ligament injuries, including partial tears.”
Oae et al. 2010	34 consecutive patients needed an operation because of severe problems such as osteochondral lesions, synovitis and instability (15 chronic cases of them were included in this review.)	29 years (ranged from 13 to 55 years), *n* = 34	19 men and 15 women, *n* = 34	With a history of ankle injury of more than 4 weeks	Cohort study	2b	Stress radiography, ultrasonography, and MRI	Arthroscopic findings	“We obtained satisfactory results with US and MR imaging.”
Park et al. 2012	48 people suspected of chronic ankle ligament injury	36 years (ranged from 19 to 64 years)	25 men and 23 women	The duration of symptoms ranging from several months to several years	Cohort study	2b	MRI	Surgical findings	“In conclusion, MRI does not show perfect sensitivity for the evaluation of chronic lateral ankle ligament injury, such as those to the ATFL and CFL. Diagnosis of a complete tear of the ATFL on MRI is more sensitive than the diagnosis of a complete tear of the CFL. MRI findings of CFL injury were diagnostically specific but were not sensitive.”
Staats et al. 2017	30 patients with CAI and failed conservative treatment	39.1 years (ranged from 18 to 71 years)	15 men and 15 women	Not mentioned	Cohort study	2b	MRI	Arthroscopic findings	“MRI is a helpful tool for preoperative evaluation, but arthroscopy remains gold standard in the diagnosis of associated lesions in patients with CAI”
Sugimoto et al. 2002	37 ft of 35 patients in whom recurrent instability of the ankle was diagnosed	29.1 years (ranged from 11 to 56 years)	16 men and 19 women	Mean interval between the injury and arthrography was 4 years 3 months (range, 6 months–22 years)	Cohort study	2b	Subtalar arthrography, manual anterior drawer test and talar tilt test	Surgical findings	“Subtalar arthrography is valuable in making the diagnosis of calcaneofibular ligament injury in recurrent instability of the ankle.”
Tan et al. 2017	82 patients with ankle inversion trauma history (42 in the chronic group were included in this review.)	24.8 years (ranged from 17 to 48 years), *n* = 42	36 men and 6 women, *n* = 42	Interval from time of injury to time of MRI more than 3 months	Cohort study	2b	MRI	Surgical findings	“MRI was able to accurately diagnose lateral ankle ligament tears in most cases. Diagnosis of a complete ATFL tear on MRI is more sensitive than that of complete CFL tear. The MRI findings of CFL injury were diagnostically specific but were not sensitive.”

### Methodological quality assessment

**Fig. 2:**
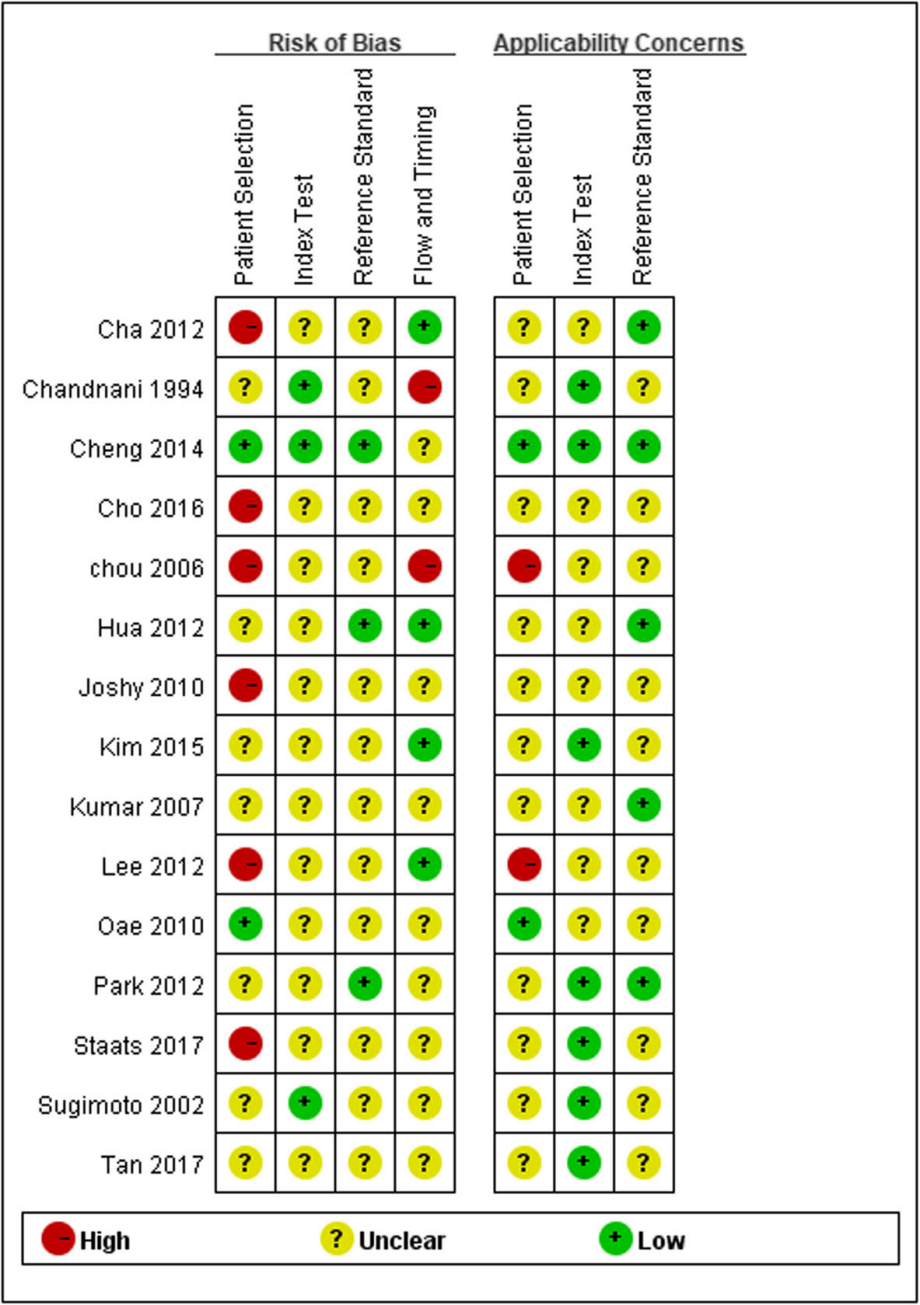
Methodological quality assessment of included studies using QUADAS-2 tool. Red stands for high risk. Green stands for low risk. Yellow stands for unclear risk

Methodological quality assessment was conducted for each study using QUADAS-2 tool (Fig. 2). All studies mentioned that participants were diagnosed of chronic ligament injury, of which 9 studies [36–38, 42, 43, 45, 46, 48, 49] mentioned the time from initial ankle inversion trauma to study commencement or duration when patients presented with chronic symptoms. Six of 15 studies [35, 38, 39, 41, 44, 47] were categorized as high risk of bias due to patient selection. All studies mentioned arthroscopic or surgical findings as their reference standards, of which 3 studies [37, 40, 46] mentioned that the reference standard results were interpreted without knowledge of the results of the index tests. All studies mentioned that investigators were blind to arthroscopic or surgical findings when interpreting the diagnostic imaging.

### Diagnostic accuracy of imaging techniques

**Fig. 3:**
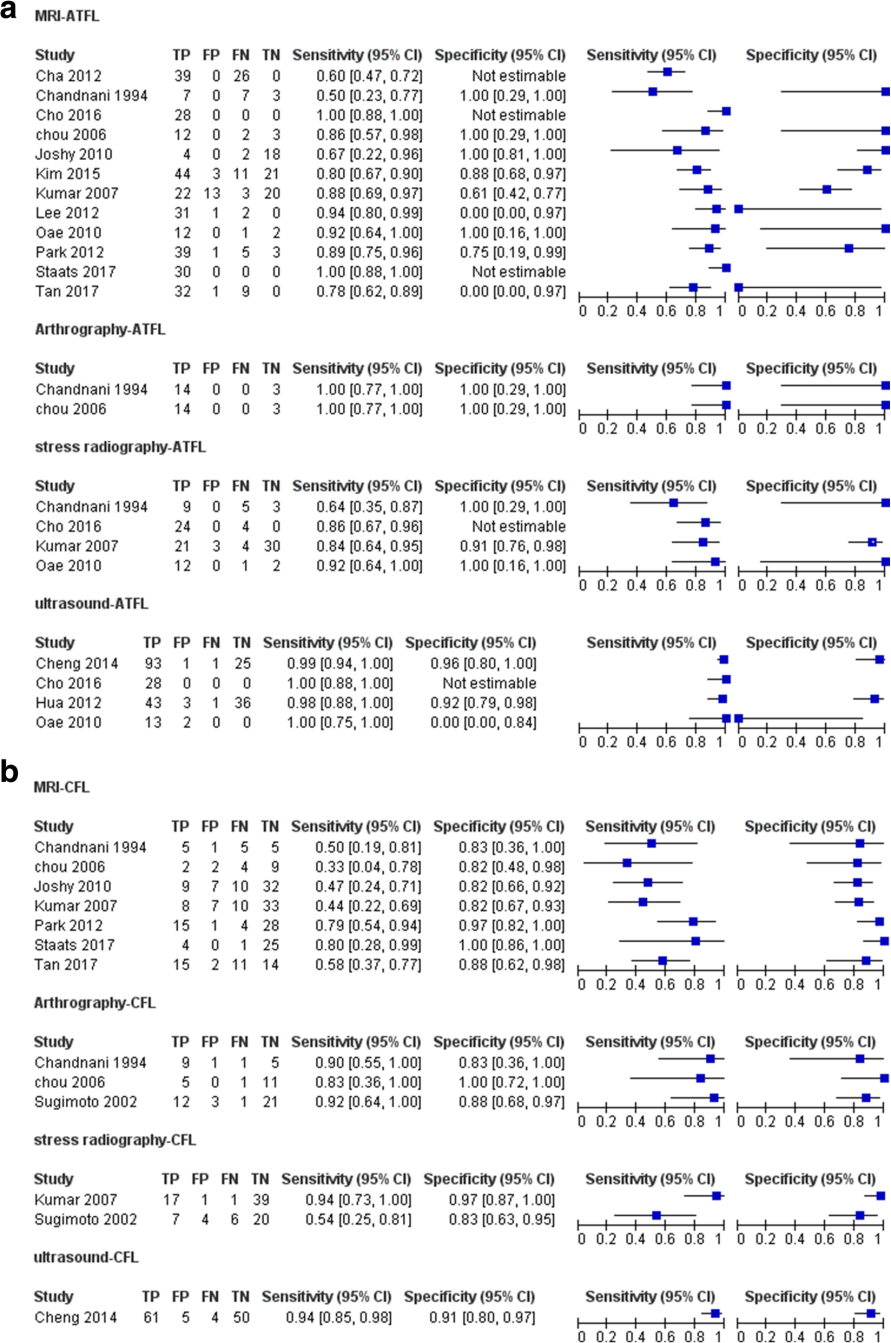
Forest plot showing sensitivity and specificity for each included study. MRI, US, stress radiography, and arthrography studies for ATFL and CFL are listed in **a** and **b**. TP, true positive; FP, false positive; FN, false negative; TN, true negative

**Fig. 4:**
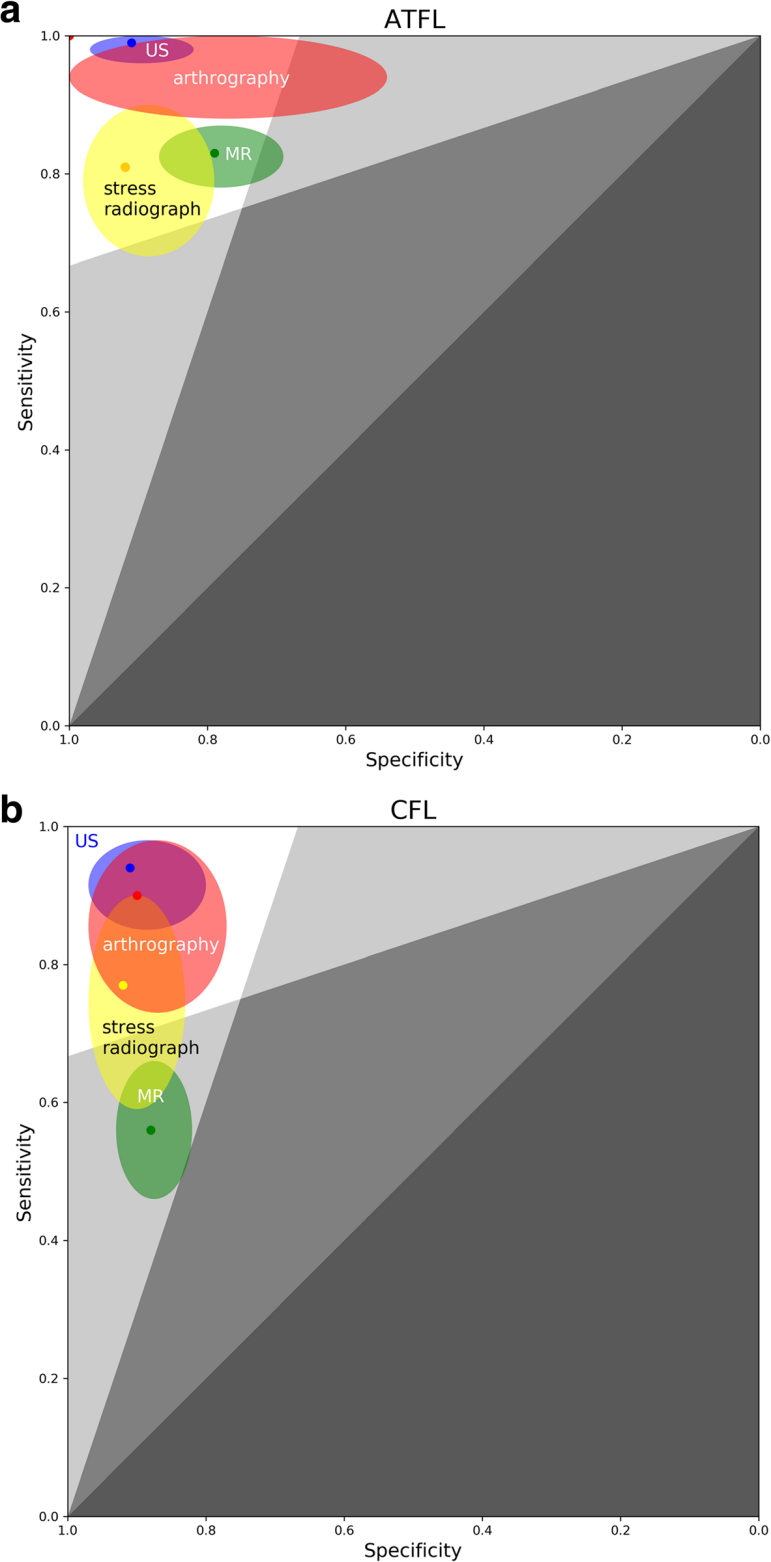
Zones of mostly bad imaging efficacy (ZOMBIE) plot for ATFL (**a**) and CFL (**b**). This is based on the receiver operating characteristic (ROC) plot. The colored dot stands for pooled sensitivity and specificity of each imaging technique, and the colored oval stands for 95% confidence interval of each imaging technique (green, MR; blue, US; red, arthrography; yellow, stress radiograph). Upper-left boomerang-shaped area formed by white and light grey zones defines zone of acceptable efficacy. Upper arm of the boomerang-shaped area stands for negative likelihood ratio below 0.33. Left arm of the boomerang-shaped area stands for positive likelihood ratio above 3

Considering the combination of different ligaments (ATFL and CFL) and imaging techniques (MRI, US, stress radiography, and arthrography), 8 subgroups were analyzed. Extracted data of 8 subgroups are listed in Fig. 3. Pooled sensitivity, pooled specificity, and their 95% confidential interval are listed in Fig. 4 in the form of zones of mostly bad imaging efficacy (ZOMBIE) plot as Richardson [50] described. Detailed pooled data are listed in Table 3.

**Table 3:** Results of pooled data

Test	Ligament	Studies	Participants	TP	FP	FN	TN	Pooled sensitivity		Pooled specificity		Pooled LR+		Pooled LR−		Pooled DOR	
								Value [95%CI]	*I* ^2^	Value [95%CI]	*I* ^2^	Value [95%CI]	*I* ^2^	Value [95%CI]	*I* ^2^	Value [95%CI]	*I* ^2^
MRI	ATFL	12	457	300	19	68	70	0.83 [0.78, 0.87]	50.4%	0.79 [0.69, 0.87]	68.6%	2.72 [1.41–5.24]	60.4%	0.28 [0.19–0.41]	20.7%	16.25 [7.65–34.53]	0.0%
MRI	CFL	7	269	58	20	45	146	0.56 [0.46, 0.66]	30.5%	0.88 [0.82, 0.93]	48.9%	3.84 [1.99–7.40]	36.5%	0.55 [0.41–0.74]	38.8%	8.28 [3.08–22.24]	48.7%
US	ATFL	4	246	177	6	2	61	0.99 [0.96, 1.00]	0%	0.91 [0.82, 0.97]	71.6%	15.08 [5.85–38.87]	0%	0.02 [0.00–0.07]	0%	946.51 [159.33–5622.80]	0%
US	CFL	1	120	61	5	4	50	0.94 [0.85, 0.98]	–	0.91 [0.80, 0.97]	–	10.3 [4.5–23.9]	–	0.07 [0.03–0.18]	–	153 [7–3550]	–
stress radiography	ATFL	4	118	66	3	14	35	0.81 [0.68, 0.90]	44.9%	0.92 [0.79, 0.98]	0%	7.94 [3.11–20.28]	0%	0.24 [0.11–0.54]	46%	39.24 [10.50–146.56]	0%
stress radiography	CFL	2	95	24	5	7	59	0.77 [0.59, 0.90]	86.6%	0.92 [0.83, 0.97]	75.7%	9.88 [0.65–149.20]	83.8%	0.20 [0.01–4.14]	89.3%	53.40 [0.50–5752.7]	88.2%
Arthrography	ATFL	2	34	28	0	0	6	1.00 [0.88, 1.00]	0%	1.00 [0.54, 1.00]	0%	–	–	–	–	–	–
Arthrography	CFL	3	70	26	4	3	37	0.90 [0.73, 0.98]	0%	0.90 [0.77, 0.97]	26.5%	7.55 [3.15–18.05]	0%	0.15 [0.06–0.39]	0%	69.85 [13.77–354.41]	0%

*Abbreviation*: *US* ultrasonography, *ATFL* anterior talofibular ligament, *CFL* calcaneofibular ligament, *TP* true positive, *FP* false positive, *FN* false negative, *TN* true negative, *LR+* positive likelihood ratio, *LR−* negative likelihood ratio, *DOR* diagnostic odds ratio

MRI exhibited the pooled sensitivities of 0.83 [0.78, 0.87] in diagnosing chronic ATFL injury and 0.56 [0.46, 0.66] in diagnosing chronic CFL injury. The pooled specificities were 0.88 [0.82, 0.93] in diagnosing chronic CFL injury and 0.79 [0.69, 0.87] in diagnosing chronic ATFL injury.

US presented the pooled sensitivities of 0.99 [0.96, 1.00] in diagnosing chronic ATFL injury and 0.94 [0.85, 0.98] in diagnosing CFL injury. The pooled specificities were 0.91 [0.82, 0.97] in diagnosing chronic ATFL injury and 0.91 [0.80, 0.97] in diagnosing chronic CFL injury.

Stress radiography demonstrated a sensitivity of 0.81 [0.68, 0.90] and a specificity of 0.92 [0.79, 0.98] in diagnosing chronic ATFL injury. Two studies regarding stress radiography for CFL showed high heterogeneity [43, 48], with *I*^2^ for pooled data above 50%.

Sample size regarding arthrography in diagnosing chronic ATFL injury is relatively small, with only 34 subjects included. Arthrography presented the pooled sensitivity of 0.90 [0.73, 0.98] and the pooled specificity of 0.90 [0.77, 0.97] in diagnosing chronic CFL injury.

## Discussion

Accurate diagnosis of chronic lateral ankle ligament injury is considered critical for surgical intervention of chronic ankle instability [13]. Imaging diagnosis is usually non-invasive and can be of value when physical tests are ambiguous [16]. Results for the four including imaging diagnostic techniques have been reported; however, the diagnostic accuracy of each imaging technique is variable in different studies, partly due to different reference standards applied [20, 21]. Our study set arthroscopic or surgical findings as the gold standard to investigate four imaging techniques. This gold standard reduced the heterogeneity of different studies.

In our results, the pooled sensitivities and specificities of US in diagnosing chronic ATFL and CFL injury were all above 0.90. When diagnosing chronic ankle instability, another systemic review involving 6 articles reported sensitivity of US ranged from 84.6 to 100% and specificity of US ranged from 90.9 to 100% [20]. In agreement with this systemic review, our results demonstrated US is an effective imaging technique in diagnosing chronic lateral ankle ligament injury.

US can precisely discriminate different ligamentous conditions, such as torn, lax, or thickened ligaments [40]. Comparing US results of healthy people and patients with chronic ankle instability, Liu et al. reported the differences in the thickness of ATFL [51] and Croy et al. reported differences in the length of ATFL [52]. Lee et al. recommended stress US over regular one for diagnosis of chronic lateral ankle ligament injury [53]. The study of Cho et al., which was included in the current review [38], utilized stress US. Other studies included in the current review did not utilize stress US technique, but still presented high diagnostic accuracy. However, US is of limited value in assessing bone or cartilage and highly experience-reliant and may be much less efficacious in less experienced hands. Moreover, only 4 studies [37, 38, 40, 45] included in this systemic review were related to the US examination of chronic lateral ankle ligament injuries, of them only one study [37] involved CFL injuries. Only two included studies [38, 45] compared US with MRI findings and reported similar diagnostic effectiveness. US is an effective imaging technique in diagnosing chronic lateral ankle ligament injury, yet more studies are warranted to compare US with other imaging techniques.

In our results, MRI exhibited the pooled sensitivities of 0.83 in diagnosing chronic ATFL injury and 0.56 in diagnosing chronic CFL injury. The pooled specificities were around 0.8 in diagnosing chronic ATFL and CFL injury. In clinical practice, MRI is highly recommended in diagnosing ligamentous injuries [54]. Also, MRI was reported to be effective in diagnosing intra-articular lesions of chronic ankle instability, including osteochondral lesions of talus, syndesmotic injuries, and impingement syndromes [55, 56]. Using MRI, Tao et al. [57] reported more cartilage lesions in patients with combined injuries of the ATFL and CFL, compared to patients with only ATFL injury. A study showed that 86.7% of the experts recommended MRI before considering surgery in chronic ankle instability patients [18]. However, according to our results, MRI did not provide the highest sensitivity or specificity in diagnosing chronic ligament injuries. It presented different patterns in diagnosing chronic ATFL and CFL injuries. The sensitivity for diagnosing chronic ATFL injury (0.83 [0.78, 0.87]) was higher than that for diagnosing chronic CFL injury (0.56 [0.46, 0.66]). MRI is still irreplaceable in assessing chronic lateral ankle ligament injury because it is frequently performed to confirm or exclude the presence of concomitant lesions and influence the precise surgical technique for a certain patient. Two included studies [38, 45] in the current systemic review compared US with MRI findings and did not report significantly different diagnostic effectiveness.

Arthroscopy was recommended as a complementary to MRI for definitive diagnosis [17, 18, 22]. An expert consensus from Guillo et al. recommended that an arthroscopy should be performed at the time of surgery unless intra-articular pathology has been excluded by MRI scan and there is no history of pain [17].

In our results, stress radiography demonstrated a sensitivity of 0.81 and a specificity of 0.92 in diagnosing chronic ATFL injury. Tourné et al. suggested that stress radiography presented high specificity (up to 100%) but low sensitivity (57%), suggesting dynamic radiographs only have diagnostic value if they are positive [58]. According to our results, stress radiography showed similar high specificity as US. When diagnosing chronic ATFL injury, the sensitivity of stress radiography is still above 0.80, and the pooled LR− is 0.24 [0.11–0.54], similar as that of MRI. Negative stress radiographic findings decreases the post-test probability of chronic ATFL and CFL injury in patients with ankle inversion trauma history. However, ligamentous laxity detected through stress radiography is not synonymous with chronic ankle instability [59]. Large variability in talar tilt and anterior drawer stress radiography precludes their routine use in diagnosing chronic ankle instability [60].

The role of anesthesia in stress radiography was reported by McCaskie et al. [61], suggesting larger discriminative capability under anesthesia. Amongst studies included in the current review, only one study reported stress radiography under anesthesia [43]. The large heterogeneity between stress radiography results for chronic CFL injury may partly explained by anesthetic condition. Though stress radiography is helpful in the diagnosis, it is hardly a useful tool in predicting surgical outcomes. Jeong et al. reported stress radiography-positive patients were not statistically significantly different from stress radiography-negative patients in prognostic features [62].

Arthrography is indicated for staging and detecting intra-articular lesions of chronic lateral ankle instability [63]. Samoto et al. reported that patients with combined injuries of the ATFL and CFL diagnosed through arthrography had worse prognosis than patients with only ATFL injury [64]. Arthrography is an invasive imaging technique. With the spread of arthroscopy, which is also invasive but much more accurate, this technique is now losing its popularity. Only 3 studies before year 2010 discussed arthrography [36, 39, 48].

There are several limitations in the current systemic review. First, 6 of the 15 included studies were graded as high risk of bias due to patient selection. Unlike meta-analysis of clinical intervention, in meta-analysis of diagnostic tests, it is common to include case-control studies considered as high risk of bias [65]. Case-control studies create a preselected patient population and should be interpreted with caution. Second, associated lesions of chronic lateral ankle ligament injury were not discussed in the current review; however, these associated lesions spotted on images would certainly affect the judgement of clinicians. Third, some studies compared the diagnostic accuracy of identical imaging technique with different parameters and/or configurations on diagnosing chronic lateral ankle ligament injury. Strength of the MRI machines varied among different studies. This diversity in configuration may cause the heterogeneity within each subgroup in this study. Moreover, the size of the included studies was relatively small. Of the 15 included studies, only a total of 695 participants were included. Studies reporting the imaging diagnosis of PTFL injury are not sufficient to draw meaningful conclusion. The studies comparing different imaging techniques for the same group of subjects are limited. This suggests more diagnostic studies with high quality are warranted for imaging diagnosing chronic lateral ankle ligament injury.

## Conclusion

This systematic review with meta-analysis investigated the accuracy of imaging for the diagnosis of chronic lateral ankle ligament injury. Our results demonstrated that ultrasound manifested high diagnostic accuracy in diagnosing chronic lateral ankle ligament injury. MRI presented the diagnostic sensitivity and specificity around 0.8 for chronic ATFL injuries, but much lower sensitivity for chronic CFL injuries. Clinicians should be aware of the limitations of MRI in detecting chronic CFL injuries. Stress radiography showed similar high specificity as US in diagnosing chronic lateral ankle ligament injury and showed diagnostic value of both positive and negative results. Arthrography demonstrated sensitivity and specificity around 0.9 in diagnosing chronic CFL injury, but limited literature recommended arthrography due to invasiveness, especially in recent years. When the diagnosis of chronic lateral ankle ligament injury is uncertain after careful inquiry of the patient’s history and physical tests, US may be a helpful imaging technique in experienced hands.

## Abbreviations

ATFL: Anterior talofibular ligament
CENTRAL: Cochrane Central Register of Controlled Trails
CFL: Calcaneofibular ligament
DOR: Diagnostic odds ratio
FN: False negative
FP: False positive
LR: Likelihood ratio
MRI: Magnetic resonance imaging
PTFL: Posterior talofibular ligament
QUADAS-2: Revised Quality Assessment of Diagnostic Accuracy Studies
TN: True negative
TP: True positive
US: Ultrasonography
ZOMBIE plot: Zones of mostly bad imaging efficacy plot
